# Endogenous Oscillations Time-Constrain Linguistic Segmentation: Cycling the Garden Path

**DOI:** 10.1093/cercor/bhab086

**Published:** 2021-05-05

**Authors:** Lena Henke, Lars Meyer

**Affiliations:** Research Group Language Cycles, Max Planck Institute for Human Cognitive and Brain Sciences, 04103 Leipzig, Germany; Research Group Language Cycles, Max Planck Institute for Human Cognitive and Brain Sciences, 04103 Leipzig, Germany; Clinic for Phoniatrics and Pedaudiology, University Hospital Münster, 48149 Münster, Germany

**Keywords:** delta-band oscillations, P600, segmentation, sentence comprehension

## Abstract

Speech is transient. To comprehend entire sentences, segments consisting of multiple words need to be memorized for at least a while. However, it has been noted previously that we struggle to memorize segments longer than approximately 2.7 s. We hypothesized that electrophysiological processing cycles within the delta band (<4 Hz) underlie this time constraint. Participants’ EEG was recorded while they listened to temporarily ambiguous sentences. By manipulating the speech rate, we aimed at biasing participants’ interpretation: At a slow rate, segmentation after 2.7 s would trigger a correct interpretation. In contrast, at a fast rate, segmentation after 2.7 s would trigger a wrong interpretation and thus an error later in the sentence. In line with the suggested time constraint, the phase of the delta-band oscillation at the critical point in the sentence mirrored segmentation on the level of single trials, as indicated by the amplitude of the P600 event-related brain potential (ERP) later in the sentence. The correlation between upstream delta-band phase and downstream P600 amplitude implies that segmentation took place when an underlying neural oscillator had reached a specific angle within its cycle, determining comprehension. We conclude that delta-band oscillations set an endogenous time constraint on segmentation.

## Introduction

Language comprehension requires listeners to decode linguistic information before speech acoustics fade from working memory. This challenge may be overcome by segmenting sentences into multiword units of limited duration (Christiansen and Chater 2016). Segmentation is thought to expand memory capacity, prolong storage intervals, and thus maximize efficiency (Kurby and Zacks 2008).

While segmentation may mitigate memory limitations, the duration of segments is still constrained. Findings from various domains suggest a 3-s window for the integration of information (Pöppel 1997, 2009; Wittmann 2011; Christiansen and Chater 2016). For instance, the canonical working memory limit of 4–7 items (Miller 1956; Cowan 2001) translates to a duration of 2–3 s (Baddeley et al. 1975). In time perception research, events were found to be perceived as simultaneous only when sharing an interval of roughly 3 s (Fraisse 1984; Pöppel 1997); likewise, subjects could reproduce temporal intervals only up to a duration of about 3 s, beyond which accuracy declined (Elbert et al. 1991; Ulbrich et al. 2007). For language, a window of six words has been proposed (Frazier and Fodor 1978), which translates to about 2.4 s when assuming an average speech rate of 150 words per minute (Tauroza and Allison 1990). Likewise, utterances in spontaneous speech have a median duration of 2.6 s (Vollrath et al. 1992).

Electroencephalography research on language comprehension suggests that such duration constraints could reflect the periodicity of the underlying electrophysiological activity (Roll et al. 2012; Schremm et al. 2015). Roll et al. (2012) presented participants with sentences consisting of three clauses (e.g., *Martin cuts delightedly the grass*/*so it is short*/*when he has time*). Presentation rate was adjusted to fit each clause, two clauses, or all three clauses into a single time window of 2.7 s. Only when a clause ended at a multiple of 2.7 s, a Closure Positive Shift (CPS) was elicited, an event-related brain potential (ERP) associated with the termination of a multiword unit (Steinhauer et al. 1999; for review, see Bögels et al. 2011a, 2011b). In general, prolonged durations increase the likelihood for segmentation and a CPS (Hirose 2003; Swets et al. 2007; Hwang and Schafer 2009; Hwang and Steinhauer 2011; Webman-Shafran et al. 2015).

Research on neural oscillations at delta-band frequency (i.e., <4 Hz—that is, cyclic electrophysiological potential changes in the order of seconds) further supports the notion of periodic electrophysiological activity as temporal constraint. Ding et al. (2015) investigated entrainment to syntactic structure, finding that delta-band oscillations are in synchrony with syntactic phrases. Likewise, Meyer et al. (2016) found that delta-band phase predicts phrase termination. These effects appear to be independent of the processing of prosodic markings that indicate segmentation acoustically (Frazier et al. 2006; Kreiner and Eviatar 2014).

We hypothesized that delta-band oscillations are the neural substrate of time constraints on segmentation. We recorded EEG while subjects listened to ambiguous sentences (e.g., *Yesterday, the conductor interrupted the flutist and the drummer delighted the listener.*). By manipulating speech rate, we aimed to bias segmentation: At a slow rate, *the flutist* ended with a 2.7-s window; a segmentation time window of 2.7 s would thus yield the correct segmentation. In contrast, a fast rate would create a so-called garden path, squeezing *the flutist and the drummer* into a 2.7-s window. A segmentation time window of 2.7 s would yield the wrong segmentation; at *delighted*, this should trigger a P600, an ERP elicited in the context of syntactic violations or unexpected sentence continuations (Hagoort et al. 1993; Osterhout et al. 1994; Dröge et al. 2016; Kuperberg et al. 2019). If the resulting segmentation fault results from oscillatory activity, the phase angle of the delta-band oscillation at the offset of *the flutist* should predict P600 amplitude at *delighted*. To control for the undesired effect of prosody on segmentation, we orthogonally manipulated the presence of acoustic boundary cues (e.g., Frazier et al. 2004, 2006; Snedeker and Casserly 2010). In sum, the present study aims at extending prior evidence for temporal constraints on sentence segmentation from the visual to the auditory modality while controlling as much as possible for prosodic factors; we also hope to enrich the discussion about the underlying neural mechanism.

## Materials and Methods

### Participants

Forty-eight native German speakers participated in the study (24 female; mean age = 24.61 years, standard deviation (SD) = 3.34 years). One additional participant only attended the first of two experimental sessions (see below) and was thus excluded. Participants were right handed (Oldfield 1971; mean lateralization quotient = 89, SD = 13.70) and reported no history of neurological, hearing, or language disorder. The local ethics committee of the University of Leipzig approved the study (file 060/17-ek) to be consistent with the declaration of Helsinki. Written informed consent was obtained from participants before participation.

### Materials

We used German sentences containing a temporary ambiguity (e.g., *Yesterday, the conductor interrupted the flutist and the drummer delighted the listener.*; Hoeks et al. 2006). At the offset of *the flutist*, listeners could continue the current segment, resulting in the noun phrase segmentation (NP) *the flutist and the drummer* (a). Under the alternative clause segmentation (CL), *the drummer* would start the new segment *the drummer delighted the listener* (b). Critically, under the NP segmentation, the following verb *delighted* cannot be syntactically integrated with *the drummer* and elicits a so-called garden path, requiring reinterpretation (Frazier and Rayner 1982; Hoeks et al. 2002, 2006; Frazier 2016). In contrast, the CL segmentation leads to the correct structure, with no reinterpretation required. Note that at *delighted*, the English translation could still be interpreted as a passive, deferring ambiguity resolution until *the listener*. This interpretation is, however, not possible for the original German stimuli.

**Figure 1:**
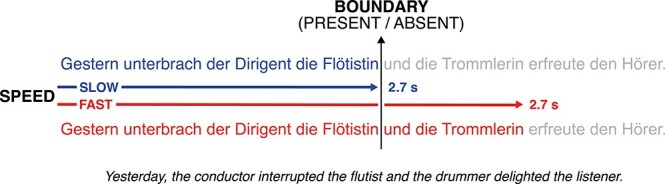
Overview of experimental manipulations.

(a) [Yesterday, the conductor interrupted the flutist and the drummer]…
(b) [Yesterday, the conductor interrupted the flutist] and [the drummer…

The factorial 2 × 2 design used the factors RATE (FAST versus SLOW) and BOUNDARY (PRESENT versus ABSENT) to dissociate time constraints from prosody (see Fig. 1). We aimed to elicit the contrasting segmentations (a) and (b) via FAST and SLOW speech rates: At the FAST rate, the first clause including *the flutist and the drummer* fell into a single time window of 2.7 s from sentence onset. This aimed at eliciting the NP segmentation and thus the garden-path effect. In the SLOW condition, 2.7 s ended after *the flutist*, aimed at eliciting the correct CL segmentation. To assess whether a time constraint or prosody triggered segmentation, an intonational phrase boundary (IPB) was either PRESENT or ABSENT at the offset of *the flutist*, thus a segment could be terminated or continued.

Sixty sentence exemplars were created, matched for total syllable number and number of syllables before the offset of *the flutist*. Log lemma frequencies of the verbs and nouns in all positions (dlexDB; Heister et al. 2011) were normally distributed (Shapiro–Wilk test, *W* > 0.97, *P* > 0.12) and showed no outliers according to the interquartile criterion. To prevent noun semantics from affecting segmentation, the two nouns surrounding the word *and* were counterbalanced and matched for syllable number and frequency within sentence (paired t-test; *t*(59) = −0.62, *P* = 0.54). Nouns were morphologically marked for female gender, because of the syncretism of the feminine nominative and accusative case in German that was required to elicit the coordination ambiguity. For each counterbalanced version, the PRESENT and ABSENT conditions were recorded at a natural speech rate by a professional male speaker (mean duration of unmanipulated audio files *M* = 5.53 s, SD = 0.28, corresponding to approximately four syllables per second). Recordings were normalized to 60 dB sound-pressure level. FAST and SLOW versions were then created while preserving perceptually relevant acoustic cues (Schlueter et al. 2014) using the PSOLA algorithm (Moulines and Charpentier 1990) implemented in Praat (Boersma 2002). Manipulation factors for the FAST (*M* = 0.62, SD = 0.03) and SLOW (*M* = 0.94, SD = 0.05) conditions yielded speech rates within the intelligible range (Foulke and Sticht 1969; Beasley et al. 1980; Ghitza and Greenberg 2009). FAST and SLOW conditions were also created for control sentences to avoid inferences based on speech rate.

Efficacy of the BOUNDARY manipulation was assessed statistically on the last syllable of the word before the boundary (i.e., *−tist* of *the flutist*) and the following pause (Table 1). As an undesired side effect of the RATE manipulation, pause duration, preboundary syllable length, and pitch slope all showed interactions between RATE and BOUNDARY (nonparametric analysis of repeated-measures data; all *F*(1,476) > 26.14, all *P* < 0.001; Noguchi et al. 2012) as well as main effects of RATE (all *F*(1,476) > 276.00, all *P* < 0.001) and BOUNDARY (all *F*(1,476) > 40.40, all *P* < 0.001). A Nemenyi test showed that all boundary cues were less salient in the FAST compared to the SLOW condition (all *q* > 8.49, all *P* < 0.001). Within the FAST conditions, all cues still differed between the ABSENT and PRESENT conditions (all *q* > 5.59, all *P* < 0.001); within the SLOW conditions, only pause duration and pitch rise differed (*q* = 20.37, *P* < 0.001 and *q* = 8.56, *P* < 0.001, respectively). Because not all boundary cues always co-occur in natural speech (Peters et al. 2005), analyses suggested an audible IPB in both the FAST and the SLOW conditions. Yet, we note that the additional boundary cue in the FAST condition could counteract the hypothesized RATE-dependent garden-path effect.

**Table 1:** Acoustic analysis of boundary manipulation^*^

Boundary	Absent		Present	
Rate	Fast	Slow	Fast	Slow
Pause duration (ms)	2 ± 6	8 ± 16	214 ± 25	342 ± 43
Preboundary syllable duration (ms)	186 ± 19	261 ± 27	170 ± 18	257 ± 27
Pitch slope^**^	94 ± 145	63 ± 113	226 ± 120	153 ± 81

^*^Mean ± standard deviation

^**^Linear fit across preboundary syllable

Each pseudo-randomized stimulus list contained 60 experimental sentences in total, 15 of each condition. Additionally, 60 control sentences ending after *the drummer*—30 of FAST and SLOW each—were included to prevent participants from always expecting a continuation of the sentence and thus choosing the correct segmentation by strategy. The order of the two nouns surrounding *and* as well as the levels of RATE were counterbalanced within participants between control and target sentences. Half of the control sentences were presented in the first half of the experimental session, while their matching target sentence appeared in the second half—and vice versa. To distract participants from the experimental manipulation, 40 filler sentences from a previous study unrelated to the present study were additionally included (Meyer et al. 2014). To increase signal-to-noise ratio while avoiding habituation (Branigan 2007; Pickering and Ferreira 2008), each participant completed two experimental lists (resulting in a total of 120 experimental sentences) each at a different visit in our laboratory with a break of at least 1 week (mean duration = 10.17 days, SD = 4.24 days). All experimental and control variables (i.e., order of the nouns, RATE, BOUNDARY and part of list) were counterbalanced across the experimental lists for the two sessions and the order of these lists was counterbalanced across participants.

To ensure continued attention, participants were asked to answer a two-alternative forced-choice comprehension question after each sentence (e.g., *Did the conductor interrupt the drummer?*). The question was targeted at the ambiguous region of the sentence (i.e., where *the drummer* could be interpreted as part of the NP or CL) aiming to uncover the segmentation (Christianson et al. 2001). Hence, when participants encounter the garden path (i.e., interpret *the drummer* as NP), we expect a high number of incorrect *yes* answers to the target sentences. Given that the correct answer to this question is *no* for all target sentences and *yes* for all control sentences, we changed 20% of the questions for each sentence type requiring a different response.

### Procedure

Participants listened to the sentences in an acoustically shielded cabin. Stimuli were presented using Presentation® (Neurobehavioral Systems, Inc., Albany, US). Each trial started with a green fixation cross (1500 ms), which transitioned to red (500 ms) for stimulus playback. Participants were instructed to fixate the cross at all times and to blink during green crosses to reduce artifacts. A jitter interval (mean duration = 249 ms, range: 0–500 ms) preceded playback to avoid coupling between sensory modalities, as visual cues can phase-reset electrophysiological activity in the auditory cortex (Lakatos et al. 2008; Power et al. 2012). Playback was followed by a 2-s buffer interval to safeguard unbiased estimation of delta-band phase during sentence comprehension. Subsequently, the comprehension question was presented visually together with the two answer options beneath. Participants were instructed to answer as quickly and accurately as possible. Timeout was 3 s. Order of answer choices was counterbalanced within conditions. Participants performed 10 additional trials at the beginning of the experiment covering all sentence types and manipulations to familiarize with the procedure. An entire session, including short breaks and the fitting of the EEG cap, took approximately 2 h.

### Data Acquisition

The EEG was recorded continuously from 63 Ag/AgCl electrodes mounted in an elastic cap (ANT Neuro GmbH, Berlin, DE) according to the extended international 10–20 system. Vertical and horizontal eye movements were monitored by bipolar electrodes on the outer canthi of both eyes as well as below and above the right eye. The setup was referenced on-line to the left mastoid (A1) and an additional electrode on the stratum served as the ground. The EEG signal was sampled at 500 Hz using a TMSi Refa8 amplifier in combination with the QRefa Acquisition Software (Max Planck Institute for Human Cognitive and Brain Sciences, Leipzig, DE). Impedances were kept below 10 kΩ.

### Data Analysis

Response accuracy was analyzed in R (R Core Team 2019) by fitting a logistic mixed-effects model with the contrast coded fixed effects RATE and BOUNDARY with interaction term. As a random effect, we entered intercepts for subjects and items, which resulted in the maximal converging model. P-values were obtained by likelihood ratio tests of the full model against a reduced model without the effect in question.

For EEG preprocessing, we adapted the Harvard Automated Preprocessing Pipeline (HAPPE; Gabard-Durnam et al. 2018), combining EEGLAB functions (Delorme and Makeig 2004) and custom MatLab® (The MathWorks, Inc., Natick, US) code. To facilitate automated artifact removal using independent component analysis (ICA; Makeig et al. 1996), raw continuous data were downsampled to 250 Hz and high-pass filtered with a 1-Hz two-pass 6th-order Butterworth infinite impulse response (IIR) filter. Bad channels were identified by means of the normed joint probability of the average log power and rejected if surpassing an outlier threshold of 3 SD (mean number of removed channels = 3.70, SD = 1.38). A wavelet-enhanced ICA was applied to remove large artifacts for optimal component classification in the next step. Then, MARA (Winkler et al. 2011) was used to detect artifact components. Components with an artifact probability >0.5 were rejected (mean number of rejected components = 23.11, SD = 6.78). Data were re-referenced to the common average of all scalp electrodes excluding the channels marked as bad, which were spherically interpolated. To achieve a neutral reference across conditions, data were then re-referenced to an approximate zero reference (REST; Dong et al. 2017). After preprocessing, one participant was excluded from further analysis because no artifact components were identified in the data of one experimental session, yet visual inspection clearly indicated substantial noise.

**Figure 2:**
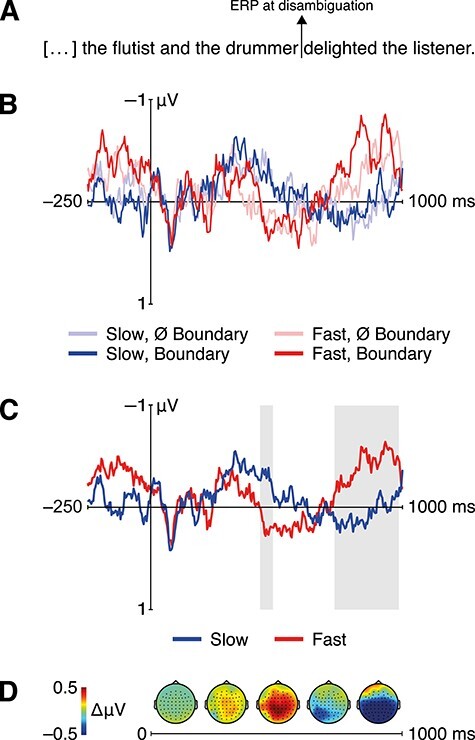
ERP results with a baseline from 0 to 150 ms. (*A*) English translation of experimental stimulus with marker for ERP analysis time point. (*B*) Grand-average ERPs elicited by the disambiguating verb for all conditions. (*C*) Grand-average across levels of factor rate; gray shadings indicate time windows of significant clusters. (*D*) Topographic maps representing scalp distribution of the ERP difference between FAST and SLOW conditions (200-ms windows for illustration only).

For statistical analysis, we used the FieldTrip package (Oostenveld et al. 2011). To investigate ERPs at disambiguation, the preprocessed data were epoched into trials from −0.5 to 1.5 s around the onset of the disambiguating verb (e.g., *delighted*). A standard baseline window from −200 to 0 ms violated the assumption that there are no differences between conditions in the baseline window. Based on the RATE manipulation, there was indeed different auditory input within this time window, which would have forwarded baseline differences between the SLOW and FAST conditions into a spurious condition difference (cluster-based permutation within the time window from −200 to 0 ms of the factor RATE; cluster-sum *t*(46) < −983, cluster-level *P* < 0.01). Therefore, the baseline correction employed an interval from 0 to 150 ms into the disambiguating verb (Hwang and Steinhauer 2011). Correction effectively aligned sensory components across conditions (Fig. 2*B*). ERPs were analyzed in a factorial fashion, averaging across trials within participants across sessions crossing RATE and BOUNDARY. Statistical analysis employed cluster-based permutation testing within the time window from 0.15 to 1 s after the onset of the disambiguating verb excluding the baseline interval, aiming to identify significant time–electrode clusters while controlling for false positives (Maris and Oostenveld 2007; two-sided, α = 0.05, 10 000 permutations, ≥3 channels minimum cluster size).

For analysis of delta-band phase during segment termination (i.e., around *the flutist*), data were downsampled to 100 Hz and low-pass filtered with a 4-Hz two-pass 10th-order Butterworth IIR filter. Analytic phase was derived by the Hilbert transform. Epochs from −3 to 3 s around the critical segmentation point were created to avoid edge artifacts while allowing for low-frequency analysis. To assess whether phase during segmentation predicted the ERP at disambiguation, we used circular–linear correlation analysis (Fisher 1993; Berens 2009). First, within participant, across trials, at each electrode and each sample from −0.5 to 0 s around the segmentation point, we correlated delta-band phase with the amplitude of the EEG during disambiguation, masked for the time point and electrode at which the ERP peaked (i.e., the P600; see Results). Correlation coefficients underwent Fisher z-transformation (Fisher 1915) and were then compared to a surrogate distribution based on correlation values from 10 000 permutations of randomly reassigned trials; FDR-correction was used to control for false positives (Chakravarthi and VanRullen 2012; Zoefel et al. 2019).

## Results

### Behavioral Results

All participants understood the task, as indicated by high accuracy on comprehension questions to both filler (mean accuracy = 98.16%, SD = 13.45%) and control sentences (mean accuracy = 94.69%, SD = 22.42%). Due to experimenter error, six participants had a prolonged timeout in their first experimental session. To assess whether this affected performance on the target sentences, we first fitted their data with a logistic mixed-effects model that included only the fixed effect SESSION (first versus second) and random intercepts for subjects and items. We compared the fit of this model to the fit of a reduced model without the factor SESSION. Model comparison was not significant (*P* = 0.08); hence, these participants were kept for group analysis. The logistic mixed-effects model over all participants indicated neither an effect of RATE (*P* = 0.06), BOUNDARY (*P* = 0.33), nor an interaction of both factors (*P* = 0.57) on the response accuracy to the comprehension questions.

### Electrophysiological Results

ERPs at disambiguation (i.e., at *delighted*, Fig. 2*A*) showed a positivity for the FAST compared to the SLOW condition (cluster-sum *t*(46) = 1864, cluster-level *P* = 0.002; peak at CP1, 0.456 s) from 0.43 to 0.49 s over the entire scalp (Fig. 2*C*). Additionally, there was a negativity for the FAST as compared to the SLOW condition (cluster-sum *t*(46) = −10 060, cluster-level *P* < 0.001; peak at P2, 0.856 s) from 0.73 to 0.99 s. Neither an interaction with BOUNDARY nor a main effect of BOUNDARY was observed (all *P* > 0.34 and *P* > 0.06, respectively). To control whether this pattern resulted from our nonstandard baseline interval, we reran the analysis using a baseline of −200 to 0 ms in a time window from 0 to 1 s after the onset of the disambiguating verb. This alternative baseline prolonged the positivity (0–0.17, 0.18–0.72 s) and shortened the negativity (0.83–0.87 s).

**Figure 3:**
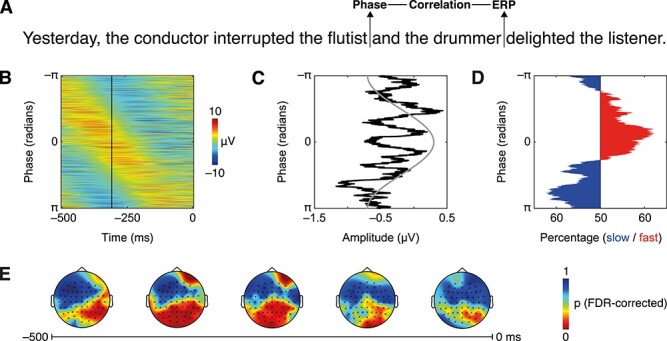
Phase results. (*A*) English translation of experimental stimulus with marker for analysis time points. (*B*) Phase at electrode CP1 in the vicinity of critical segmentation point, all trials of all participants sorted by phase; black line indicates time point of correlation peak. (*C*) EEG amplitudes at disambiguation sorted by phase angle during segmentation, overlaid on a single cosine cycle. (*D*) Percentage of trials per condition. (*E*) Topography of FDR-corrected p-values. For illustration only, (*C*) and (*D*) are smoothed by a moving window of 500 trials.

Delta-band phase immediately prior to the segmentation point (i.e., the offset of *flutist*; −0.48 to −0.21 s) significantly correlated with the EEG amplitude masked for the peak of the positive ERP cluster (mean Fisher’s *z* = 0.196, *P* < 0.05, FDR corrected; peak at CP1 electrode, −0.456 s; Fig. 3), but not the negative ERP cluster (all *P* > 0.77, FDR corrected).

### Control Analyses

Based on a reviewer’s concern, we wanted to ensure that the observed ERP effect actually was a garden-path effect. To this end, we conducted a supplementary online study. To monitor the garden-path effect at the critical word, we used a self-paced reading task that has previously been shown sensitive to garden-path effects (Frazier 1987; Hoeks et al. 2006). We created an unambiguous baseline for each sentence by exchanging the conjunction *and* for *but* (German: *doch*; e.g., *Yesterday, the conductor interrupted the flutist but the drummer delighted the listener.).* We additionally included an adverbial phrase (e.g., *on the stage*) at the end of the control sentences in order to avoid differences in reading times based on sentence length (i.e., when a sentence continued as compared to when it ended). Because the unambiguous replacement of *but* would have lead to an ungrammatical control sentence, we created a version that included the adverbial phrase at the same position as the ambiguous control sentence, yet, also kept the remainder of the sentence at the end. Based on the null effect of prosody in the EEG study, we only employed the factor AMBIGUITY (i.e., *and* versus *but*) and focused our analysis on its main effect within the target sentences. We created 48 experimental lists with 120 sentences each (30 sentences per condition) counterbalanced according to the same criteria as the EEG experiment. Participants read the sentences by pressing the space bar on their keyboard to advance from one word to another. To ensure that they read the sentences attentively, they were additionally asked a comprehension question after each sentence. The delay of question presentation was 500 ms. Note that the participants of the self-paced reading study also participated in an additional auditory control experiment. This experiment aimed to control for the possibility that in the EEG study, the extended pause between the auditory sentence stimulus and the comprehension question—which we introduced for unbiased phase estimation—may have diluted the garden-path effect in the comprehension task. In the same manner as the self-paced reading study, we only employed the factor RATE (i.e., FAST versus SLOW). In contrast to the EEG study, we confined the delay of the question presentation to 500 ms. Order of participation was counterbalanced between the two experiments.

We recruited 48 participants (all right handed; 13 females; mean age = 27.94 years, SD = 4.91 years) using Prolific (www.prolific.co) and tested them on the online platform Gorilla (Anwyl-Irvine et al. 2020). Application of the interquartile criterion to participants’ mean accuracy over both experiments resulted in the exclusion of two participants from statistical analysis. One additional participant was excluded due to more than 50% missed responses in one of the experiments. To analyze comprehension performance, we fitted logistic mixed-effects models with the fixed effect AMBIGUITY or RATE—depending on the experiment—and random effects for subject and item only on the target sentences. Replicating the behavioral results from the EEG experiment, there was no significant difference between the full and the reduced model in either of the experiments (*P* > 0.16), indicating that the absence of a garden-path effect in the EEG experiment was not simply due to the timing of the comprehension question. To test for the garden-path effect during self-paced reading, we analyzed reading times at the disambiguating verb. Reading times were log-transformed to achieve a normal distribution and outliers in the target conditions were removed according to the interquartile criterion. A linear mixed-effects model on the target sentences with the fixed effect AMBIGUITY and the random effects participant and item showed a significant effect of AMBIGUITY (χ^2^(1) = 16.94, *P* < 0.001) with longer reading times for the ambiguous (*M* = 424.89 ms, SD = 172.16) as compared to the unambiguous sentences (*M* = 406.60 ms, SD = 162.50). In line with previous findings, we interpret the prolonged reading times in the ambiguous condition as a garden-path effect. Note also that order of participation in the auditory and visual experiment did not influence the reading times from the self-paced reading study when added as a fixed effect to the model (*P* = 0.15).

A reviewer of our original manuscript raised the possibility that instead of a garden-path effect, the positivity observed in the EEG study could reflect differences in speech rate alone. To address this, we compared the difference wave between the FAST and SLOW conditions at the disambiguating verb across the significant electrodes and time window of the positivity to the difference waves at each other content word position. We excluded the sentence-initial word because of the sentence onset response. We employed the same postonset baseline from 0 to 0.15 s. A series of cluster-based permutation tests showed a statistically significant difference at each content word position (all cluster-sum *t*(46) > 422.58, all cluster-level *P* < 0.01), suggesting that the ERP at the disambiguating word does not reflect differences in speech rate alone.

Lastly, we were concerned that the correlation between delta-band phase and downstream single-trial EEG amplitude could be confounded by differences in prosody induced by the different speech rates and according differences in boundary strength (Table 1). Delta-band oscillations can entrain to prosody (Bourguignon et al. 2013; Gross et al. 2013; Mai et al. 2016) and prosodic boundaries trigger segmentation (Frazier et al. 2006; Snedeker and Casserly 2010; Kreiner and Eviatar 2014; Ghitza 2017, 2020). To test this, we calculated coherence between the pitch envelope and the single-trial delta-band signal at the correlation peak electrode across frequencies from 0 to 4 Hz on a data segment the duration of a full delta cycle (i.e., −1.5–1.5 s) centered at the peak time point within the FAST and SLOW conditions. As trial numbers did not differ across conditions, we did not correct coherence for distributional bias (Bokil et al. 2007). Given the expected differences in peak frequencies of the acoustic signal due to the rate manipulation, we averaged coherence across frequencies within participant and compared coherence between conditions. There was no significant difference in coherence between conditions (Shapiro–Wilk test; *W* > 0.94, *P* > 0.03; Wilcoxon signed-rank test; *z*(46) = −0.61, *P* = 0.54). Together with the absence of a main effect of BOUNDARY in the ERP, this likely indicates that prosody entrainment was not the main cause for the observed effects.

## Discussion

We hypothesized that delta-band oscillations underlie the previously reported 2.7-s time constraint on segmentation during language comprehension. Likelihood of incorrect syntactic interpretation of a sentence increased when an ambiguous word was included in a time window of 2.7 s. This is indexed by the P600 upon disambiguation, indicating that listeners had expected a particular segmentation pattern that was then falsified by the incoming verb. This is agnostic to any specific functional interpretation of the P600, which has been previously proposed to reflect cognitive operations such as the revision of an initial syntactic analysis or the global sentence interpretation, a prediction error, or the reranking of states in a ranked-parallel architecture (Osterhout et al. 1994; Kaan and Swaab 2003; Bornkessel and Schlesewsky 2006; Kuperberg 2007; Levy 2008; Chang and Fitz 2014; Dröge et al. 2016; Hale et al. 2018; Kandylaki and Bornkessel-Schlesewsky 2019; Kuperberg et al. 2019). This interpretation is also consistent with our self-paced–reading result. After the P600, we also observed a broadly distributed negativity. Such negativities have previously been reported for incorrect sentence continuations (Steinhauer et al. 2010; Steinhauer and Drury 2012; Hampton Wray and Weber-Fox 2013) and under increases in working memory demands (Ruchkin et al. 1992; Kluender and Kutas 1993; Mecklinger et al. 1995; Fiebach et al. 2001, 2002; Phillips et al. 2005). Accordingly, we suggest that the observed late negativity could be an index of increased memory demands. During the revision of the segmentation, the unintegrated words need to be kept in working memory (Steinhauer et al. 2010). Out of the two ERPs observed here, only the P600 correlated with the phase of the delta-band oscillation. Based on this result and our hypothesis, we will focus on the P600 in the following.

Delta-band phase at the time point where a segment was either continued or terminated predicted P600 amplitude on the single-trial level. This suggests that oscillatory activity with cycle durations in the range of seconds—indexed by the phase of oscillations below 4 Hz—is the neural substrate of time constraints on syntactic segmentation (Vollrath et al. 1992; Hirose 2003; Swets et al. 2007; Hwang and Schafer 2009; Hwang and Steinhauer 2011; Roll et al. 2012; Schremm et al. 2015; Webman-Shafran et al. 2015). This converges on the prior observation that delta-band oscillations align with to-be-decoded syntactic structure (Ding et al. 2015; Meyer et al. 2016; Bonhage et al. 2017). Yet, the present data leave an involvement of semantic processing unclear. While Bonhage et al. (2014) could not dissociate whether delta-band oscillations reflected syntactic or semantic chunking, the time constraint was found not to influence semantic processing in prior behavioral studies (Schremm et al. 2016). Further investigation is required to extend the present research questions to semantic processing.

We here claim that slow-frequency oscillations set an endogenous time constraint on segmentation. However, an alternative explanation for the present findings is the decay of short-term memory, which has been associated with the 2.7-s constant in language and verbal processing in general (Wingfield and Byrnes 1972; White 2017). At the moment, we may only hypothesize that the segmentation account and the short-term memory account are not mutually exclusive, such that cycles of 2.7 s duration could also be the units of the memory buffer that is employed in sentence comprehension. In analogy, such a buffer could work like the phase–amplitude-coupled theta- and gamma-band oscillations in the working memory architecture by Lisman and Jensen (2013). In this architecture, theta-band cycles are associated with the binding of multiple to-be-memorized items, which are individually represented by gamma-band cycles replayed at specific theta-band phase segments (Axmacher et al. 2010; Kaminski et al. 2011; Friese et al. 2012; Vosskuhl et al. 2015). It would be interesting to test for an analogous relationship between segmentation-related delta-band oscillations and a faster frequency band that represents individual syllables or words.

The current findings suggest that endogenous oscillatory time constraints can trigger segmentation independently of prosodic boundaries (Meyer et al. 2016). This provides a new perspective on the classical observation that internal segmentation affects auditory perception (Fodor and Bever 1965; Garrett et al. 1966). In particular, prosodic boundaries are more salient perceptually when coinciding with the boundaries of syntactic constituents (Buxó-Lugo and Watson 2016). This endogenous constraint is likely established during ontogenesis: While segmentation in infants requires prosodic boundaries, these become more and more obsolete after 6 years of age (Männel and Friederici 2009; Männel et al. 2013; Wiedmann and Winkler 2015). Our result adds to the discussion about an involvement of endogenous oscillations in comprehension (Meyer et al. 2020a, 2020b; Ghitza 2020; Giraud 2020; Haegens 2020; Kandylaki and Kotz 2020; Klimovich-Gray and Molinaro 2020; Lewis 2020). Specifically, we may suggest that segmentation must not always rely on exogenous entrainment to prosody but can also reflect endogenous processing cycles that act as pacemaker.

We certainly acknowledge that our manipulation did induce prosodic differences between the SLOW and FAST conditions. Still, prosody entrainment (e.g., Bourguignon et al. 2013) did not differ between the FAST and SLOW conditions, which were in the intelligible range (Foulke and Sticht 1969; Beasley et al. 1980; Ghitza and Greenberg 2009). Additionally, we did not obtain an interaction effect on the ERP at disambiguation, which should have occurred if the stronger boundary in the SLOW condition had aided segmentation more strongly than the weaker boundary in the FAST condition. In line with this, we cannot entirely rule out a possible effect of the RATE manipulations on the ERP that is not induced by entrainment. For instance, the negativity in the time window before verb onset (i.e., in the standard baseline window) might—similarly to the negativity following the P600—reflect a sustained negativity that has previously been found as an index of increased working memory demands (Ruchkin et al. 1992; Fiebach et al. 2001, 2002). In the present study, this could have been induced by the FAST speech, where more words needed to be kept in memory within a single time unit. Using this time window as a baseline hence shifts this difference in the ERP, resulting in a positivity that starts almost immediately at word onset. However, our control analysis has shown that the observed positivity stands comparison to other words that underwent the very same rate manipulation. Therefore, it is unlikely that the positivity on the disambiguating verb purely stems from RATE differences and associated memory demands.

We suggested that delta-band oscillations time-limit the segmentation of speech into multiword units. But what type of units? Potential candidates are 1) *prosodic phrase*—a stretch of speech flanked by prosodic boundaries (e.g., Pierrehumbert 1980); 2) *implicit prosodic phrase—*a prosodic phrase without overt prosodic boundaries (e.g., in reading; for review, see Breen 2014); 3) *syntactic phrase*—a sequence of words that functionally depend on each other (e.g., determiner and noun in *the conductor* in (b); e.g., Bresnan and Kaplan 1982); 4) *constituent*—a syntactic phrase that serves a syntactic function as a whole (e.g., the object *the flutist and the conductor* in (a); Carnie 2001; cf. Osborne and Niu 2017); 5) *segment*—a stretch of speech that has been identified as a unit based on prosody or statistics (e.g., Soderstrom et al. 2003); and 6) *chunk*—a segment that is converted to an abstract level (Abney 1991; Christiansen and Chater 2016). Out of these terms, we suggest that *implicit prosodic phrase*, *constituent*, *segment*, and *chunk* are compatible with our findings, because they describe cognitive rather than perceptual units. *Syntactic phrases* are likely too anisochronous to imply an oscillatory generator (Meyer et al. 2020a, 2020b).

While we labeled the ERP at disambiguation a P600, it could also be a CPS—or a combination of both: In the FAST condition, participants supposedly interpreted the sentence as an NP segmentation until disambiguation. Correspondingly, the termination of this segment would coincide with the end of an interval of 2.7 s and could hence elicit a CPS (Roll et al. 2012). On the one hand, the P600 and the CPS have similar topographies, and the comparably early peak of the observed positivity would converge on the observation that the CPS peaks before the P600 (Steinhauer 2003). On the other hand, P600 latency is notorious for its variability (e.g., fuelling the so-called P600-as-P300 hypothesis; Sassenhagen et al. 2014; Sassenhagen and Fiebach 2019). Nevertheless, a CPS at disambiguation in the FAST condition would still indicate a segmentation fault. Note that this interpretation would further support the proposal that the CPS in the average ERP is the time-domain equivalent of a delta-band phase reset (Sauseng et al. 2007; Meyer et al. 2016, 2020b). We encourage future research on this.

Finally, the assumption of a 2.7-s time window for segmentation is likely too static to claim ecological validity. While prior literature suggests a limited segmentation window with a duration somewhere within the 2- to 3-s range (Baddeley et al. 1975; Pöppel 1997; Roll et al. 2013; Schremm et al. 2015), this window is likely flexible within and variable across subjects. Indeed, prior work has found segment duration in sentence processing to correlate with working memory capacity (Swets et al. 2007; Roll et al. 2012, 2013; Mccauley and Christiansen 2015). Future work should assess this variability in more detail and link it to the range of frequency-domain electrophysiological variability.

## Conclusions

Our findings suggest that time constraints on sentence segmentation have a periodic electrophysiological substrate: Cycles of endogenous delta-band oscillations may time-limit the segmentation of speech into multiword units, whereby the phase angle of the delta-band oscillator enforces segmentation every 2.7 s.

## Funding

This work was supported by the Max Planck Society through the award of the Max Planck Research Group *Language Cycles* to Lars Meyer.

## Notes

We thank Heike Boethel for data acquisition and two anonymous reviewers for their helpful comments on a previous version of this manuscript. *Conflict of Interest*: none declared.
