# Correction to: Endogenous oscillations time-constrain linguistic segmentation: cycling the garden path

**DOI:** 10.1093/cercor/bhac497

**Published:** 2022-12-02

This is a correction to: Lena Henke, Lars Meyer, Endogenous oscillations time-constrain linguistic segmentation: cycling the garden path, *Cerebral Cortex*, Volume 31, Issue 9, September 2021, Pages 4289–4299, https://doi.org/10.1093/cercor/bhab086.

In the originally published version of this manuscript, there was an error in the documentation of the high-pass filter.

The erroneous sentence was removed: “For actual component removal, the raw data were high-pass filtered with an 0.1-Hz two-pass 4th-order Butterworth IIR filter (Winkler et al. 2015) and downsampled to 250 Hz; bad channels determined above were removed from these data as well.”

This error has now been corrected online.

